# Risk factors for increased immune reconstitution in response to *Mycobacterium tuberculosis* antigens in tuberculosis HIV-infected, antiretroviral-naïve patients

**DOI:** 10.1186/s12879-017-2700-6

**Published:** 2017-09-06

**Authors:** Tatiana Pereira da Silva, Carmem Beatriz Wagner Giacoia-Gripp, Carolina A. Schmaltz, Flavia Marinho Sant’Anna, Maria Helena Saad, Juliana Arruda de Matos, Julio Castro Alves de Lima e Silva, Valeria Cavalcanti Rolla, Mariza Gonçalves Morgado

**Affiliations:** 1 Laboratory of AIDS and Molecular Immunology, Oswaldo Cruz Institute, Fundação Oswaldo Cruz (FIOCRUZ), Rio de Janeiro, Brazil; 2 Clinical Research Laboratory on Mycobacteria - National Institute of Infectious Diseases Evandro Chagas (FIOCRUZ), Rio de Janeiro, Brazil; 3 Clinical Research Laboratory on Health Surveillance and Immunization - National Institute of Infectious Diseases Evandro Chagas (FIOCRUZ), Rio de Janeiro, Brazil; 4 Platform for Clinical Research - National Institute of Infectious Diseases Evandro Chagas (FIOCRUZ), Rio de Janeiro, Brazil; 5 Laboratory of Cellular Microbiology, Oswaldo Cruz Institute, Fundação Oswaldo Cruz (FIOCRUZ), Rio de Janeiro, Brazil

**Keywords:** Tuberculosis, AIDS, Immune response, ELISpot, IRIS

## Abstract

**Background:**

Little is known regarding the restoration of the specific immune response after combined antiretroviral therapy (cART) and anti-tuberculosis (TB) therapy introduction among TB-HIV patients. In this study, we examined the immune response of TB-HIV patients to *Mycobacterium tuberculosis* (*Mtb*) antigens to evaluate the response dynamics to different antigens over time. Moreover, we also evaluated the influence of two different doses of efavirenz and the factors associated with immune reconstitution.

**Methods:**

This is a longitudinal study nested in a clinical trial, where cART was initiated during the baseline visit (D0), which occurred 30 ± 10 days after the introduction of anti-TB therapy. Follow-up visits were performed at 30, 60, 90 and 180 days after cART initiation. The production of IFN-γ upon in vitro stimulation with *Mtb* antigens purified protein derivative (PPD), ESAT-6 and 38 kDa/CFP-10 using ELISpot was examined at baseline and follow-up visits.

**Results:**

Sixty-one patients, all ART-naïve, were selected and included in the immune reconstitution analysis; seven (11.5%) developed Immune Reconstitution Inflammatory Syndrome (IRIS). The *Mtb* specific immune response was higher for the PPD antigen followed by 38 kDa/CFP-10 and increased in the first 60 days after cART initiation. In multivariate analysis, the variables independently associated with increased IFN-γ production in response to PPD antigen were CD4^+^ T cell counts <200 cells/mm^3^ at baseline, age, site of tuberculosis, 800 mg efavirenz dose and follow-up CD4^+^ T cell counts. Moreover, the factors associated with the production of IFN-γ in response to 38 kDa/CFP-10 were detectable HIV viral load (VL) and CD4^+^ T cell counts at follow-up visits of ≥200 cells/mm^3^.

**Conclusions:**

These findings highlight the differences in immune response according to the specificity of the *Mtb* antigen, which contributes to a better understanding of TB-HIV immunopathogenesis. IFN-γ production elicited by PPD and 38 kDa/CFP-10 antigens have a greater magnitude compared to ESAT-6 and are associated with different factors. The low response to ESAT-6, even during immune restoration, suggests that this antigen is not adequate to assess the immune response of immunosuppressed TB-HIV patients.

## Background

Tuberculosis (TB) is still a challenge worldwide, and HIV-infected individuals have a 10% annual risk to develop TB [1]. The manifestations of TB in HIV-infected patients are much more severe than in immunocompetent persons and are characterized by frequent extrapulmonary and miliary forms of the disease, mycobacteremia and increased immunodeficiency, which causes a deep cellular immune suppression and the occurrence of opportunistic diseases other than TB [2, 3].

cART is recommended for TB-HIV infected patients, and it has become available in many countries in the last decade. After cART introduction, immune reconstitution with a cellular response is observed in most adherent patients.

Brazil is a country with a policy of free and universal access to cART, CD4 counts and HIV viral load (VL) measurements and a public network that is able to treat all Brazilian HIV-infected patients [4]. Efavirenz-based regimens are recommended for HIV-infected patients with TB diagnosis, particularly if they are ART-naïve. In Brazil, the daily dose of efavirenz is recommended at 600 mg, irrespective of the weight of the patient, and is combined with two nucleoside analogs or a nucleotide (tenofovir) [5]. However, the concomitant therapy with rifampicin decreases the blood concentrations of efavirenz. The FDA approved a revised label for Sustiva® (efavirenz) stating that if efavirenz is co-administered with rifampicin, then the dose of efavirenz should be increased to 800 mg in patients who weigh over 50 kg. This recommendation is based on pharmacokinetic modeling using data from several trials [6]. Little is known regarding the impact of the efavirenz dose on the immuno reconstitution of TB-HIV patients.

The response to *Mtb*-specific antigens, such as Early Secreted Antigenic Target 6 (ESAT-6), Culture Filtrate Protein 10 (CFP-10) and 38 kDa protein have been correlated with TB disease activity, bacterial load, and IFN-γ production by sensitized lymphocytes during TB [7–10]. However, the restoration of the response to specific TB antigens in TB-HIV patients upon cART and anti-TB therapy, its dynamics over time and the factors associated with magnitude of the response have not been explored. This is of great concern since morbidity and mortality are presumably associated with both decreased and increased intensity of this immune response, which leads to disseminated TB and immune reconstitution of inflammatory syndrome (IRIS), respectively.

In the present study, we examined the immune response of TB-HIV patients to different *Mtb* antigens at baseline and at 6 months after cART initiation. Moreover, we also evaluated the influence of different doses of efavirenz and identified factors associated with immune reconstitution. The recruited TB-HIV patients were participants of an ongoing clinical trial [11] comparing patients treated with two different doses of efavirenz (600 mg and 800 mg), followed for 6 months from TB diagnosis and cART initiation (30 ± 10 days later).

## Methods

### Patient enrollment and study design

Patients enrolled in this study were TB-HIV-infected patients who participated in a randomized clinical trial (RCT) conducted from April 2006 to August 2012 at the Tuberculosis Clinic of the National Institute of Infectious Diseases Evandro Chagas (INI), Fundação Oswaldo Cruz, Rio de Janeiro, Brazil. The aim of this RCT was to evaluate the efficacy and safety of the concomitant use of anti-TB regimens, including rifampicin and efavirenz-based cART, in two different doses of efavirenz (600 and 800 mg) registered at clinicaltrials.gov [11].

The cART and anti-TB therapies were prescribed according to the Brazilian Ministry of Health Recommendations at the time of the trial [12, 13], and efavirenz combined with two nucleoside analogs or a nucleoside analog and tenofovir was offered in two different doses: 600 and 800 mg according to the randomization. Patients with low adherence to cART or anti-TB therapy and more than three missing visits were excluded from this analysis.

The cART was initiated during the baseline visit (D0), which occurred 30 ± 10 days after the introduction of anti-TB therapy. Follow-up visits were performed at 30, 60, 90 and 180 days after cART initiation. Demographic and clinical data as well as blood samples were collected at the baseline and follow-up visits.

During the follow-up, patients were assessed for IRIS. IRIS is defined as a documented worsening of signs or symptoms of TB while on an appropriate anti-TB therapy and cART that cannot be explained by any other diseases, resistance to TB drugs, low adherence or by an adverse reaction [14].

All the patients who agreed to participation in the immune response study signed an informed consent form. This protocol was approved by the INI Ethical Board (CAE: 0052.0.009.000–10), which is affiliated with the Brazilian National Ethics Council (CONEP).

### CD4^+^ T cell counts and viral load evaluation

CD4^+^ T cell counts and quantification of HIV VL were performed at each visit. BD Multitest monoclonal antibodies specific for CD45^+^, CD3^+^, CD4^+^ and CD8^+^ and conjugated to PerCP, FITC, APC and PE, respectively, were used to determine absolute counts of CD4^+^ and CD8^+^ T cell subsets according to the manufacturer’s instructions (BD Biosciences, Franklin Lakes, NJ, USA). The samples were evaluated using a FACSCalibur (BD, USA) and Multiset software (BD, USA). Plasma samples were obtained by centrifugation within 4 h of blood collection and aliquots were stored at −86 °C until ready for VL determinations that were performed according to the manufacturer’s guidelines (NASBA, Organon Teknika, Boxtel, The Netherlands; branched DNA assay, Versant HIV-1 RNA 3.0, Siemens, Tarrytown, USA). The lowest established VL detection limit (LDL) was 80 copies/ml.

### Peripheral blood mononuclear cell preparation

Peripheral blood mononuclear cells (PBMCs) were obtained from heparinized whole blood and were processed immediately after blood collection. PBMCs were isolated by density gradient centrifugation using Histopaque 1077 (Sigma–Aldrich, USA), cryopreserved in 90% fetal bovine serum (FBS-Gibco, Invitrogen, USA) and 10% dimethylsulfoxide (Sigma–Aldrich, USA) and stored in liquid nitrogen to be further subjected to the ELISpot assay.

### IFN- γ ELISpot assay

Briefly, 96-well plates (Millipore, USA) were coated with capture anti-IFN-γ mAb (Diaclone, France) diluted 1/100 in PBS and incubated overnight at 4 °C. After several washes with PBS, the wells were blocked for 2 h at room temperature with complete culture medium. PBMCs were added at an input cell number of 1 × 10^5^ cells/well and were unstimulated (negative control) or stimulated with phytohemagglutinin (positive control), and with different *Mtb*-related antigens in duplicates: PPD (Statens Serum Institut-Denmark), ESAT-6 (Statens Serum Institut-Denmark) or 38 kDa/CFP-10 (kindly donated by LIONEX Immunodiagnostic GmbH, Braunschweig, Germany). All of the stimuli were used at a concentration of 5 μg/well in complete culture medium [RPMI 1640 (Sigma, USA) supplemented with 10% of FBS, Penicillin–Streptomycin (10,000 U–10,000 μg/mL), L-glutamine 200 mM, non-essential amino acids 10 mM, and sodium pyruvate 100 mM (all from Invitrogen, USA)]. After 40 h of incubation at 37 °C and a 5% CO_2_ humid atmosphere, the plates were washed 3 times with PBS, 3 times with PBS supplemented with 0.1% Tween 20 (Sigma-Aldrich) and 3 times with PBS followed by 2 h of incubation at 37 °C with 100 μl per well biotinylated detection mAb and diluted 1/100 in PBS supplemented with 1% BSA (Diaclone) (PBS 1% BSA). The wells were then washed with PBS, and 100 μl of substrate (5-bromo-4-chloro-3-indolyl phosphate/NBT; Diaclone) was added per well. The colorimetric reaction was terminated after 10 min at room temperature by washing the wells several times with water. The spots were counted using an automated ELISpot reader (CTL Analyzers LLC, Cellular Technology, USA). The results are expressed as spot-forming cells (SFC)/million PBMCs. The response was considered positive if ≥50 SFC/10^6^ PBMCs were obtained after subtraction of the mean background obtained with non-stimulated cells [15].

### Statistical analysis

Demographic characteristics and clinical, virological and immunological parameters were assessed for TB-HIV patients at baseline and during follow-up visits. Chi-squared distribution for categorical variables and the Mann-Whitney test for continuous variables were used to compare groups (included vs. excluded patients). Wilcoxon sign-rank, and McNemar tests were used to compare the immune responses, which were expressed as numerical (SFC/10^6^ PBMCs) and categorical (positive or negative) variables within patients at different study time points, respectively. We fitted a log-linear model where each follow-up visit represents one observation, and the patients were considered at the cluster-level with random effects and modeled by a working autoregressive (AR1) correlation structure. Then, the adjustment of the covariance was made, taking into account observations of the same individual and their proximity in time, although the fixed effects had been obtained through the mean value of the visits. The numerical outcome variables were the production of IFN-γ in response to 38 kDa/CFP-10 or PPD, and both were transformed into log scales for analysis. The univariable statistical analysis was made by modeling the variables one by one. The multivariable statistical analysis was made using a backward stepwise method where all of the covariates were included in the initial model but only the covariates that presented statistical significance with alpha <10% were retained in the final model. The analysis was performed using R v3.1.0 software [16].

## Results

### Study population

Eighty-eight TB-HIV patients agreed to participate in the study. Twenty-seven patients were excluded due to low adherence to HIV treatment (*n* = 9) or for attending less than three follow-up visits (*n* = 18). There were no statistically significant differences in demographic, clinical and laboratory data at baseline among the included and excluded patients (Table 1). Among the included patients, we observed a predominance of men and a similar proportion of pulmonary and disseminated TB. Moreover, median CD4^+^ T cells count were 141 cells/mm^3^ and 77% had a VL ≥ 30,000 copies/ml. IRIS incidence was 11% (7 patients).

**Table 1:** Baseline characteristics of the patients

Characteristic	Included N = 61	Excluded N =27	*P*-value
Age (Year) and median (IQR)	37	39	0.32
Gender, n (%)			0.21
Male	49(80)	25(93)	
Female	12(20)	2(7)	
Site of tuberculosis, n (%)			0.93
Pulmonary	27(44)	13(48)	
Disseminated	29(48)	12(44)	
Extrapulmonary	5(8)	2(7)	
Efavirenz dose, n (%)			0.5
600 mg	33(54)	12(44)	
800 mg	28(46)	15(56)	
IRIS, n (%)			0.1
No	54(88.52)	27(100)	
Yes	7(11.48)	0 (0)	
CD4, n(%)			0.82
< 200 cells/mm^3^	41(67)	17(63)	
≥ 200 cells/mm^3^	20(33)	10(37)	
CD4, median (IQR)	141.08(36–234)	162.74(71–227.5)	0.38
< 200 cells/mm^3^	62(31–98)	89(34–130)	
≥ 200 cells/mm^3^	302.6(234.8–355.2)	288(227.2–308)	
HIV-1 viral load (copies/ml), n (%)			1
≤ 30,000	14(23)	6(22)	
> 30,001	47(77)	21(78)	

*n* number of case, *IRIS* Immune Reconstitution Inflammatory Syndrome, *IQR* Interquartile Range

### IFN-γ producing cells in response to antigenic stimulation

**Fig. 1:**
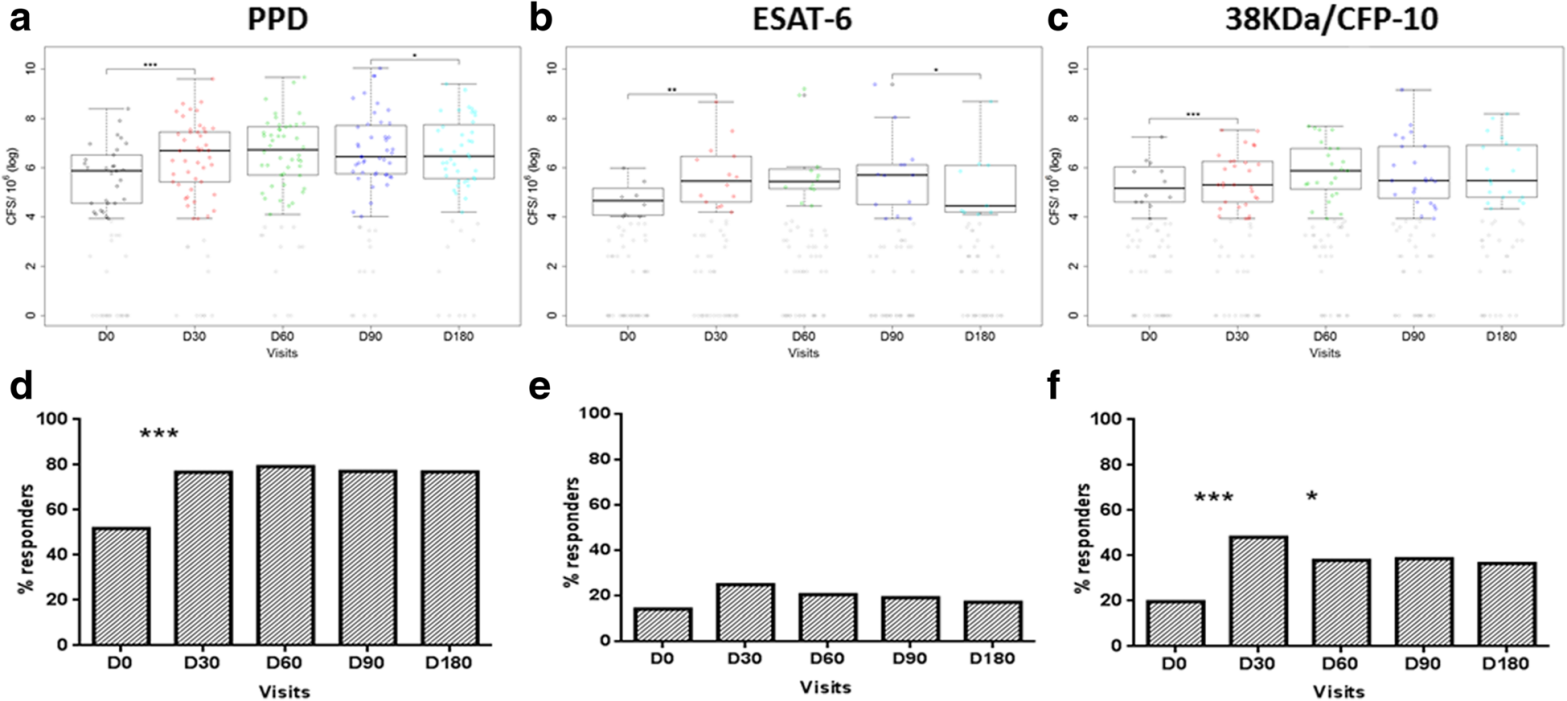
The ELISPOT assay performed in TB/HIV patients. The results of IFN-γ-producing cells in response to the specific antigens for **a** PPD, **b** ESAT-6 **c** 38 kDa/CFP-10 are expressed in log_10_ for spot forming cells (CFS). The colored points represent the magnitude of the response, and the gray points non-responders. Horizontal lines represent the median values. The statistical difference in the median number of CFS between consecutive visits was assessed through the Wilcoxon signed-rank test (***p < 0.01;**p < 0.05 and *p < 0.1). The results were also expressed as the percentage of individuals responsive to the antigens **d** PPD, **e** ESAT-6 and **f** 38 kDa/CFP-10, and individuals were considered responders with ≥50 spots/10^6^ cells. The Exact McNemar test (***p < 0.01;**p < 0.05 and *p < 0.1) was used to assess the difference of the response between consecutive visits

The ELISpot results for the presence of IFN-γ producing T cells in response to PPD, ESAT-6 and 38 kDa/CFP-10 antigens are shown in Fig. 1(a, b and c, respectively). The proportion of responders to PPD, ESAT-6 and 38 kDa/CFP-10 at baseline was approximately 49%, 14% and 20%, respectively, and increased significantly after 30 days of cART (except for ESAT-6) and then stabilized (38 kDa/CFP-10) or slightly decreased (PPD and ESAT-6) between 90 and 180 days (Fig. 1d, e and f). The proportion of responders to PPD remained higher than that for all of the other antigens during the follow-up.

### Factors associated with IFN-γ production in response to the PPD and 38 kDa/CFP-10

Multivariate analysis was conducted to identify factors associated with IFN-γ production in response to PPD (Table 2) and to 38 kDa/CFP-10 antigens (Table 3). In patients with CD4^+^ T cell counts <200 cells/mm^3^ at baseline, the mean of IFN-γ production in response to the PPD antigen was 5.547 (95% CI = [1.348; 22.821], *p* = 0.018) times that of the patients with CD4^+^ T cell counts ≥200 cells/mm^3^. Similarly, the IFN-γ production in patients aged 32 years or less was 2.808 (95% CI = [1.13; 6.98]; *p* = 0.026) times that of patients aged 38–44 years. The other variables independently associated with IFN-γ production in response to PPD were the site of TB, efavirenz dose, follow-up CD4 ≥ 200 cells/mm^3^ and follow-up CD4 < 200 cells/mm^3^ (Table 2). Regarding follow-up CD4^+^ T cell counts, it was estimated that for each increase of one unit in CD4^+^ T cell counts, the IFN-γ production in response to PPD increased 1.011 units (95% CI = [1.002; 1.02], *p* = 0.016) since CD4 counts were <200; however, when follow-up CD4^+^ T cell counts were ≥200 cells/mm^3^, the IFN-γ production in response to PPD increased 1.006 units (95% CI = [1.003; 1.009], *p* = 0.016), *p* < 0.001) for each increase of one unit in CD4^+^ T cell counts (Table 2). Further adjusting for gender did not change our results.

**Table 2:** Univariate and multivariate analyses of the factors associated with IFN-γ production in response to the PPD antigen

Factor	Univariable		Multivariable^a^	
	(IC95%)	*P*-value	(IC95%)	*P*-value
Baseline CD4				
≥ 200 cells/mm^3^	1		1	
< 200 cells/mm^3^	1.375 (0.332;5.694)	0.661	5.547 (1.348;22.821)	**0.018**
Gender				
Male	1			
Female	0.242 (0.052; 1.118)	0.069		
Age (Year)				
38–44	1		1	
32–18	3.87 (1.336;11.212)	**0.013**	2.808 (1.13;6.98)	**0.026**
37–32	1.256 (0.305;5.169)	0.752	1.061 (0.292; 3.862)	0.928
Site of tuberculosis				
Pulmonary	1		1	
Disseminated	1.173 (0.285; 4.836)	0.825	1.531 (0.468; 5.01)	0.482
Extrapulmonary	4.275 (1.122; 16.285)	**0.033**	6.431 (1.566; 26.408)	**0.010**
Ethnicity				
White	1			
Black	2.544 (0.603; 10.737)	0.204		
Mulattoes	1.376 (0.283; 6.705)	0.692		
Efavirenz dose				
600 mg	1		1	
800 mg	3.187 (0.901;11.266)	**0.072**	3.505 (1.04;11.817)	**0.043**
HIV-1 viral load				
Detectable	1			
Undetectable	2.348 (1.228;4.489)	0.010		
Follow-up CD4 cells/mm^3^				
≥200 cells/mm^3^	1.003 (1.001; 1.005)	**0.002**	1.006 (1.003; 1.009)	**<0.001**
<200 cells/mm^3^	1.004 (0.996; 1.1014)	0.312	1.011 (1.002; 1.02)	**0.016**
Follow-up %HLA-DR^+^/CD3^+^ T cells	0.994 (0.975; 1.014)	0.559		
Follow-up % CD8^+^/38^+^ T cells	0.998 (0.972; 1.024)	0.851		
Follow-up IFN-γ plasmatic	0.989 (0.946; 1.033)	0.61		

^a^The predictors in the PPD antigen final model are as follows: Baseline CD4, Age, Site of tuberculosis, Efavirenz dose, Follow-up CD4 ≥ 200 cells/mm^3^ and Follow-up CD4 < 200 cells/mm^3^

**Table 3:** Univariate and multivariate analyses of the factors associated with IFN-γ production in response 38KDa/CFP-10 antigen

Factor	Univariable		Multivariable^a^	
	(IC95%)	*P*-value	(IC95%)	*P*-value
Baseline CD4				
≥ 200 cells/mm^3^	1			
< 200 cells/mm^3^	1.189 (0.341; 4.143)	0.786		
Gender				
Male	1			
Female	0.5 (0.131; 1.9)	0.309		
Age (Year)				
38–44	1			
32–18	0.928 (0.225; 3.828)	0.917		
37–32	1.594 (0.371; 6.855)	0.531		
Site of tuberculosis				
Pulmonary	1		1	
Disseminated	1.308 (0.345; 4.954)	0.693	1.632 (0.469; 5.681)	0.441
Extrapulmonary	3.84 (0.895; 16.474)	**0.070**	3.054 (0.889; 10.494)	0.076
Ethnicity				
White	1			
Black	2.128 (0.373; 12.15)	0.396		
Mulattoes	1.29 (0.36; 4.619)	0.696		
Efavirenz dose				
600 mg	1		1	
800 mg	2.29 (0.658; 7.971)	0.193	2.765 (0.862; 8.865)	0.087
HIV-1 viral load				
Detectable	1		1	
Undetectable	2.078 (1.19; 3.627)	**0.010**	2.217 (1.258; 3.906)	**0.006**
Follow-up CD4 cells/mm^3^				
< 200 cells/mm^3^	0.999 (0.994; 1.003)	0.559		
≥ 200cells/mm^3^	1.002 (1; 1.004)	**0.015**	1.002 (1; 1.003)	**0.013**
Follow-up %HLA-DR^+^/CD3^+^ T cells	1.007 (0.993; 1.022)	0.340		
Follow-up % CD8^+^/38^+^ T cells	1.008 (0.992; 1.025)	0.327	1.017 (0.999; 1.035)	0.061
Follow-up IFN-γ plasmatic	0.995 (0.951; 1.04)	0.817		

^a^The predictors in 38 K Da/CFP-10 antigen final model are as follow: Follow-up CD4 cells/mm^3^ (≥ 200cells/mm^3^), Site of tuberculosis, Efavirenz dose, HIV-1 viral load and Follow-up % CD8^+^/38^+^ T cells

Factors associated with the production of IFN-γ in response to 38 kDa/CFP-10 were detectable HIV VL and CD4^+^ T cell counts at follow-up visits in the range ≥ 200 cells/mm^3^ with an increase of 1.002 (95% CI = [1; 1.003]; *p* = 0.013) units of IFN-γ production for each increase of one unit of CD4^+^ T cell counts. The other variables that were independently associated were efavirenz dose and follow-up % CD8^+^/38^+^ T cells (Table 3). Additionally, a trend (significance between 5% and 10%) for the association with IFN-γ production in response to 38 kDa/CFP-10 was observed for extrapulmonary TB cases compared with pulmonary TB.

### IFN-γ-producing cells in response to antigenic stimulation in IRIS and Non-IRIS Patients

**Fig. 2:**
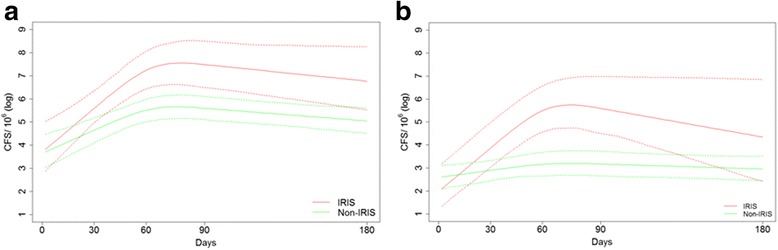
Graphic representation of the trajectories of the IFN-γ-producing cells in response to specific antigens for **a** PPD and **b** 38 kDa/CFP-10 in IRIS and non-IRIS patients during TB treatment and cART. Overall trajectories are represented as red thin lines for the IRIS group and a green thin line for the non-IRIS group. The dotted lines represent the overall 95% confidence interval for each group

The IRIS diagnosis occurred at a median of 51 days after the introduction of cART. Patients with IRIS showed an increased immune response compared with patients without IRIS, and had a marked increase in IFN-γ production by T cells in response both to PPD and, notably, to 38 kDa/CFP-10 antigens after 60 days of cART followed by a reduction, which was in contrast to patients without IRIS (Fig. 2a and b).

## Discussion

Our study is the first one to prospectively evaluate the IFN-γ production by T cells in response to *Mtb* antigens in cART-naïve TB-HIV patients before and after cART introduction using the ELISpot assay. An increase in the magnitude of response to *Mtb* antigens was observed after 30 days of cART and was higher for the PPD antigen, followed by 38 kDa/CFP-10 and ESAT-6, and then stabilized or declined later on. The proportion of responders was higher for PPD and 38 kDa/CFP-10 than for the ESAT-6 antigen. PPD was the antigen associated with the strongest response in our study and in other studies including HIV-negative TB patients [17–22]. A hypothesis to explain the higher response to PPD antigen stimulation is that it contains not only *Mtb*-specific antigens but also other cross-reactive antigens, increasing its antigenicity.

In our work, in addition to PPD, we evaluated the response to the ESAT-6 antigens separately and to the antigen CFP-10 combined with the 38 kDa. The 38-kDa protein is highly immunogenic and is antigenic for human T cells and presented a response in TB patients andindividuals exposed to *Mtb* (TST positive) [22, 23]**.** Tavares et al. (2007) demonstrated that the CFP-10 and 38 kDa antigen separately present a power of response, but when combined, this power increases in individuals with TB, which was the reference data for the choice of these combined antigens for our study [22].

The analysis of risk factors associated with an increased immune response to both antigens showed a different pattern between the PPD and 38 kDa/CFP-10 antigens. Previous studies covering Brazilian TB patients and individuals latently infected with *Mtb* showed good responses to the ESAT-6 *Mtb*-specific antigen [22, 24], which was not observed in our study. Such differences might be due to the antigenicity of this molecule and the restricted T cell repertoire of the TB-HIV patients included in our study, who were in most cases severely immunosuppressed.

In general, TB-HIV patients are immunosuppressed, and most of them do not respond well to these mycobacterial antigens even if they have TB [25]. Our interest was to study the response to these antigens after combined antiretroviral therapy in cART-naïve TB-HIV patients, assuming that immune reconstitution could improve the immune response to them. However, for ESAT-6, which is an antigen included in commercial IFN-gamma release assays to assess TB infection [26], such improvement of the immune response was not achieved, suggesting that this antigen, at least in our experience, does not seem to be adequate to evaluate the immune response in severely immunosuppressed TB-HIV patients.

However, a previous study indicated that severely immunosuppressed individuals (<200 cells/mm^3^) have a better response to PPD, ESAT-6 and CFP-10 antigens than less immunosuppressed ones (≥200 cells/mm^3^) [25]. However, our study showed that patients with CD4^+^ T cell counts <200 cells/mm^3^ at baseline exhibited an increased response to PPD but not to 38 kDa/CFP-10 compared to ones with CD4^+^ T cell counts ≥200 cells/mm^3^. Similarly, during the follow-up, increases in CD4^+^ T cell counts were associated with increased IFN-γ production in response to PPD with a steeper slope seen among those with absolute CD4^+^ T cell counts <200 cells/mm^3^ compared with those above this range, which probably reflected the unspecific and complex immune activation seen in these highly immunosuppressed patients However, IFN-γ production in response to 38 kDa/CFP-10 was associated with an increase in CD4^+^ T cell counts only among those with CD4^+^ T cell counts ≥200 cells/mm^3^, suggesting a profile of specific immune restoration in response to cART.

We have more men than women in our study, but this is an expected result since men are significantly more at risk of contracting and dying from TB than women [27]. We did not observe any association of gender with increased IFN-γ production in response to the PPD and 38 kDa/CFP-10 antigens.

Younger age (18 to 32 vs. 38 to 44 years) was associated with higher IFN-γ production in response to PPD but not 38 kDa/CFP-10. The impact of age on immune restoration stimulated by mycobacterial antigens has not yet been consistently described. Neilsen et al. (2013) observed that cells from healthy adults show an increased production of cytokines (IFN-γ, TNF, and IL-10) after stimulation with PPD when compared with children [28]. These findings highlight the potential influence of age in T cell capacity to respond to *Mtb* antigens with a possible biphasic profile since young adults apparently have a better response than both children and older adults.

In our study, the extrapulmonary forms of TB in immunosuppressed individuals were associated with a higher production of IFN-γ when T cells are stimulated with PPD antigen compared with other forms of TB. A previous study evaluating IFN-γ production in immune-compromised vs. immune-competent individuals with extrapulmonary TB failed to detect differences between the groups for ESAT-6 and CFP-10 antigens [29]. The response to the PPD antigen was not evaluated in this context, and the ESAT-6 response in our study was too low to be analyzed.

Production of IFN-γ by cells stimulated with the 38 kDa/CFP-10 antigen was associated with VL control and higher CD4^+^ T cell counts in response to cART at the follow-up. Our results might suggest that with viral control and immune reconstitution due to cART in immunosuppressed patients (TB-HIV), it is possible to restore the immune response to *Mtb*-specific antigens with increased levels of IFN-γ. This is in accordance with a previous study in TB-HIV negative patients [22].

IRIS is expected to occur in immunosuppressed individuals after cART introduction, as the immune reconstitution is more intense at the beginning of the HIV treatment to the rapid control of VL, although it is not clear why this phenomenon affects only a subset of TB-HIV patients with lower CD4^+^ T cell counts [30–32]. The low incidence of paradoxical IRIS cases in the studied population precluded the analysis of the risk factors for IRIS. However, a distinct pattern of immune response was observed in these cases, which could help in the IRIS diagnosis and contribute to the understanding of this distinct phenomenon.

Immune reconstitution is an important factor to consider when choosing the best regimen to treat TB-HIV patients. Our study was nested in a clinical trial comparing the efficacy and safety of two different doses of efavirenz (600 mg and 800 mg) in TB-HIV, cART-naïve patients. Our results showed that the patients treated with 800 mg of efavirenz had a significantly greater production of IFN-γ in response to PPD and production with borderline significance in response to 38 kDa/CFP-10. These patients showed a stronger immune restoration compared to those who received the 600 mg dose, independent of the effect on CD4^+^ T cell counts or HIV VL. Studies have been conducted to compare the antiretroviral efficacy of these two different doses, and they have both been shown to be potent in terms of viral control [33, 34]. Our findings could contribute to a better understanding of immune reconstitution and be considered in the future when choosing the best strategy to treat TB-HIV patients with efavirenz-based regimens.

The present study has some potential limitations. A larger study population would have been necessary to more accurately demonstrate, by means of more significant power, whether the association between demographic, clinical and laboratory data with IFN-γ production was significant. The low proportion of IRIS cases also prevented the exploration of risk factors for this syndrome.

These findings highlight the differences in immune response according to the specificity of the *Mtb* antigen, which contributes to a better understanding of TB-HIV immunopathogenesis.

## Conclusions

Our study demonstrated that independent of the immunosuppression stage, patients with TB-HIV who are cART-naïve developed immune restoration and VL control and improved the specific response to *Mtb* antigens after the initiation of both cART and anti-TB therapy. IFN-γ production elicited by PPD and 38 kDa/CFP-10 *Mtb* antigens have a greater magnitude compared to ESAT-6 and are associated with different factors. Indeed, the increased immune response to PPD was associated with advanced immunosuppression, young age, higher efavirenz dose and severe TB clinical forms. Moreover, higher IFN-γ secretion in response to 38 kDa/CFP-10 was associated with undetectable HIV VL and CD4^+^ T cells count at follow-up visits in the range of ≥200 cell/mm^3^. These findings contribute to the understanding of the complex phenomena of immune restoration among TB-HIV patients and give insights into the interpretation of IGRAs (Interferon Gamma Released Assays). The low response to ESAT-6, generally included in such commercial assays, even during immune restoration, suggests that this antigen is not adequate for use to assess the immune response of immunosuppressed TB-HIV patients.

## Abbreviations

APC: Allophycocyanin
BSA: Bovine serum albumin
CART: Combined antiretroviral therapy
CONEP: Brazilian national ethics council
FBS: Fetal bovine serum
FDA: U.S. food and drug administration
FITC: Fluorescein isothiocyanate
HIV: Human immunodeficiency virus
IFN-γ: Interferon-gamma
IGRA: Interferon gamma released assays
IL-10: Interleukin 10
INI: National institute of infectious diseases evandro chagas
IRIS: Immune reconstitution inflammatory syndrome
*Mtb*: *Mycobacterium tuberculosis*
PBMC: Peripheral blood mononuclear cells
PBS: Phosphate-buffered saline
PE: Phycoerythrin
PerCP: Peridinin chlorophyll
SFC: Spot-forming cells
TB: Tuberculosis
TNF: Tumor necrosis factor
VL: Viral load
